# Supplementary material for: Initiation and amplification of SnRK2 activation in abscisic acid signaling
Source: Nat Commun. 2021 Apr 28;12:2456. doi: 10.1038/s41467-021-22812-x (PMC8080645; doi:10.1038/s41467-021-22812-x)
Supplement: Supplementary file 1 — Supplementary Information [file 41467_2021_22812_MOESM1_ESM.pdf]

Initiation and amplification of SnRK2 activation in abscisic acid signaling

Lin et al.,

Supplemental Information

This file includes:

Supplementary Table 1
Supplementary Figure 1 to Figure 8 with figure legends.

Table S1. Genotypes of 103 F2 seedlings with ABA hyposensitivity.

	AGI ID	Homo	Heter	WT	χ^2	P value
RAF3	AT5G11850	101	2	0	293.23	<0.0001
RAF4	AT1G18160	61	40	2	72.72	<0.0001
RAF5	AT1G73660	89	14	0	208.41	<0.0001
RAF6	AT4G24480	8	34	61	66.44	<0.0001
RAF7	AT3G06620	62	32	7	77.85	<0.0001
RAF10	AT5G49470	34	54	15	7.85	0.0266
RAF11	AT1G67890	30	60	13	8.48	0.0148

The association between ABA hyposensitivity and RAF mutation status was estimated using Pearson's chi-squared test. Homo, number of homozygous, Heter, number of heterozygous, WT, number of wild type. Source data are provided in Source Data.

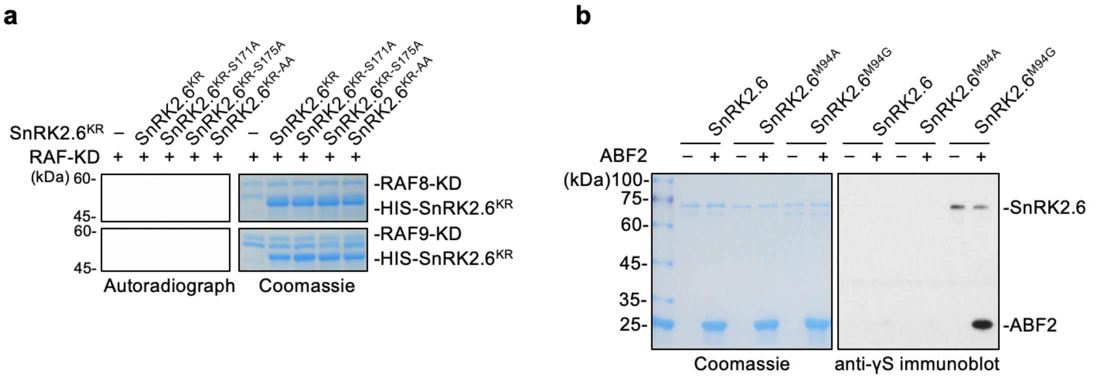

Supplementary Figure 1. RAFs phosphorylate SnRK2.6 *in vitro.*

a No detectable kinase activity of RAF8-KD and RAF9-KD *in vitro*. Recombinant RAF8-KD or RAF9-KD cannot phosphorylate wild type or mutated SnRK2.6^{KR} expressed and purified from *E. coli* in the presence of [γ-^{32}p]ATP. Autoradiograph (left) and Coomassie staining (right) show phosphorylation and loading, respectively, of purified GST-RAF-KD and HIS-SnRK2.6^{KR}. **b** SnRK2.6^{M94G}, but not SnRK2.6^{M94A} or wild type SnRK2.6, can use N^{6}-Benzyl-ATPγS to thiophosphorylate ABF2. Anti-γ-S immunoblot (right) and Coomassie staining (left) show thiophosphorylation and loading, respectively, of recombinant GST-SnRK2.6 and GST-ABF2. Images shown are representative of two independent experiments. Source data are provided in Source Data.

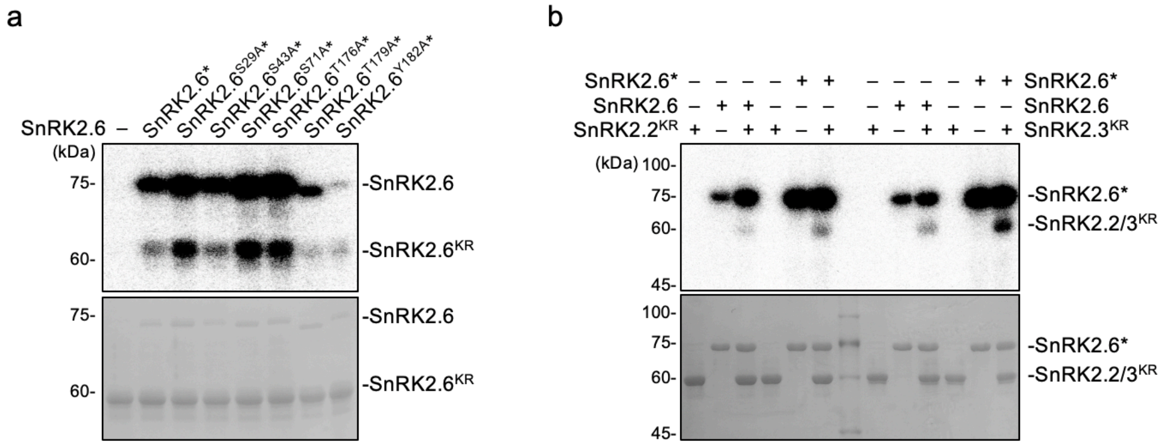

Supplementary Figure 2. Transphosphorylation of SnRK2.6 on SnRK2s.

a Transphosphorylation activity of pre-activated wild type and mutated SnRK2.6 on SnRK2.6^{KR}. Wild type and mutated SnRK2.6 were pre-incubated with HIS-SUMO-RAF10-KD coated on beads and ATP for 30 min. After removal of HIS-SUMO-RAF10-KD, pre-activated wild type and mutated SnRK2.6 were used to phosphorylate HIS-SnRK2.6^{KR}, in the presence of [γ-^{32}p]ATP. Autoradiograph (upper) and Coomassie staining (lower) show phosphorylation and loading, respectively, of purified GST-SnRK2.6 and HIS- SnRK2.6^{KR}. **b** SnRK2.6 phosphorylates SnRK2.3^{K51R} (SnRK2.3^{KR}) and SnRK2.2^{K52R}(SnRK2.2^{KR}) *in vitro*. Recombinant SnRK2.6 and HIS-SUMO-RAF10-KD pre-activated SnRK2.6 (SnRK2.6*) were used to phosphorylate HIS-SUMO-SnRK2.2^{KR} and HIS-SUMO-SnRK2.3^{KR} in the presence of [γ-^{32}p]ATP. Autoradiograph (upper) and Coomassie staining (lower) show phosphorylation and loading, respectively, of purified GST-SnRK2.6, HIS-SUMO-SnRK2.2^{KR}, and HIS-SUMO-SnRK2.3^{KR}. Images shown are representative of at least two independent experiments. Source data are provided in Source Data.

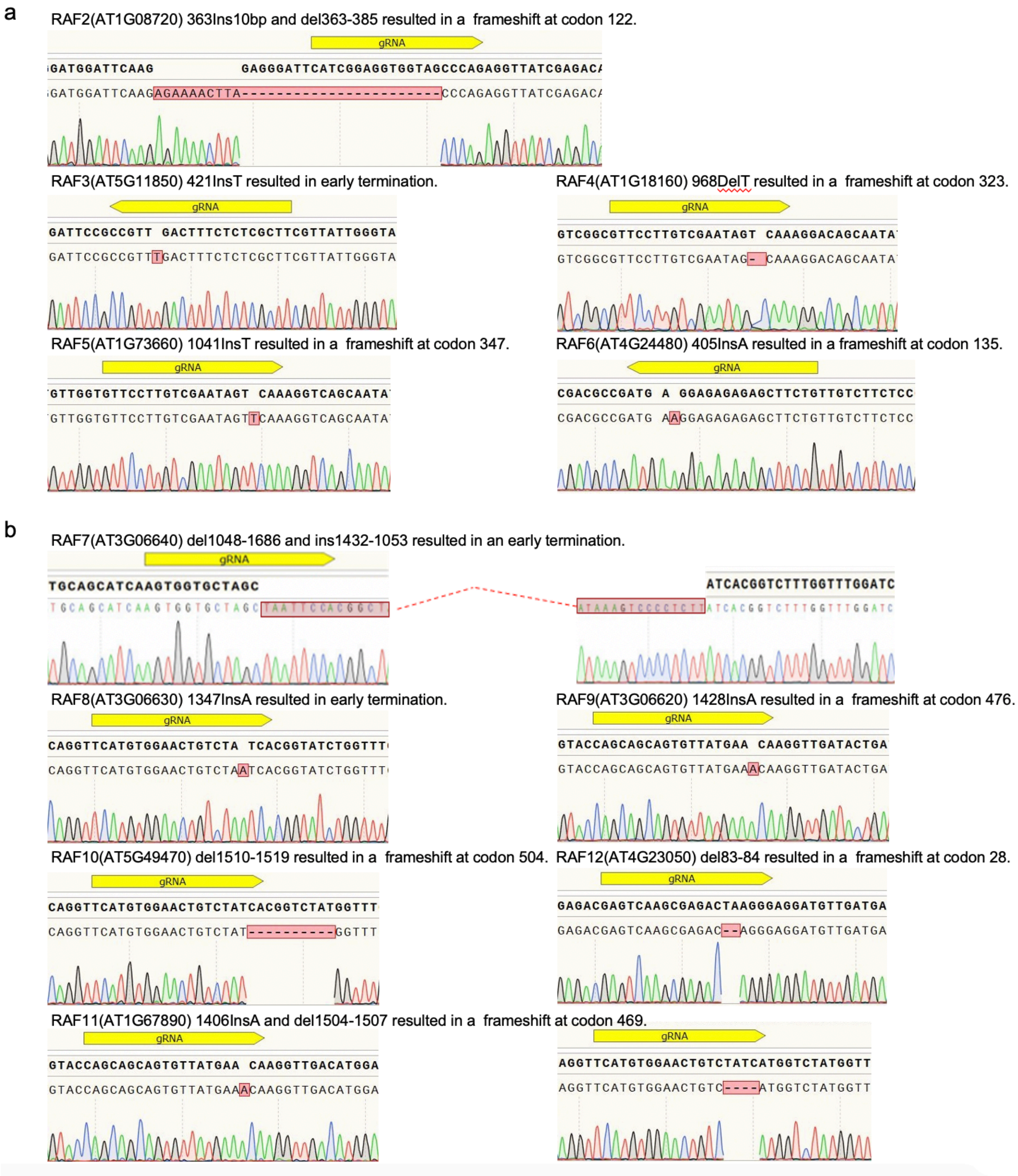

Supplementary Figure 3. Mutations of RAF genes in the OK[100]-B3 and OK[100]-B2 high-order mutants.

a Sequencing results showing the mutations in *RAF2*, *RAF3*, *RAF4*, *RAF5*, and *RAF6* genes in *OK[100]-B3* mutant. **b** Sequencing results showing the mutations in *RAF7*, *RAF8*, *RAF9*, *RAF10*, *RAF11*, and *RAF12* genes in the *OK[100]-B2* mutant.

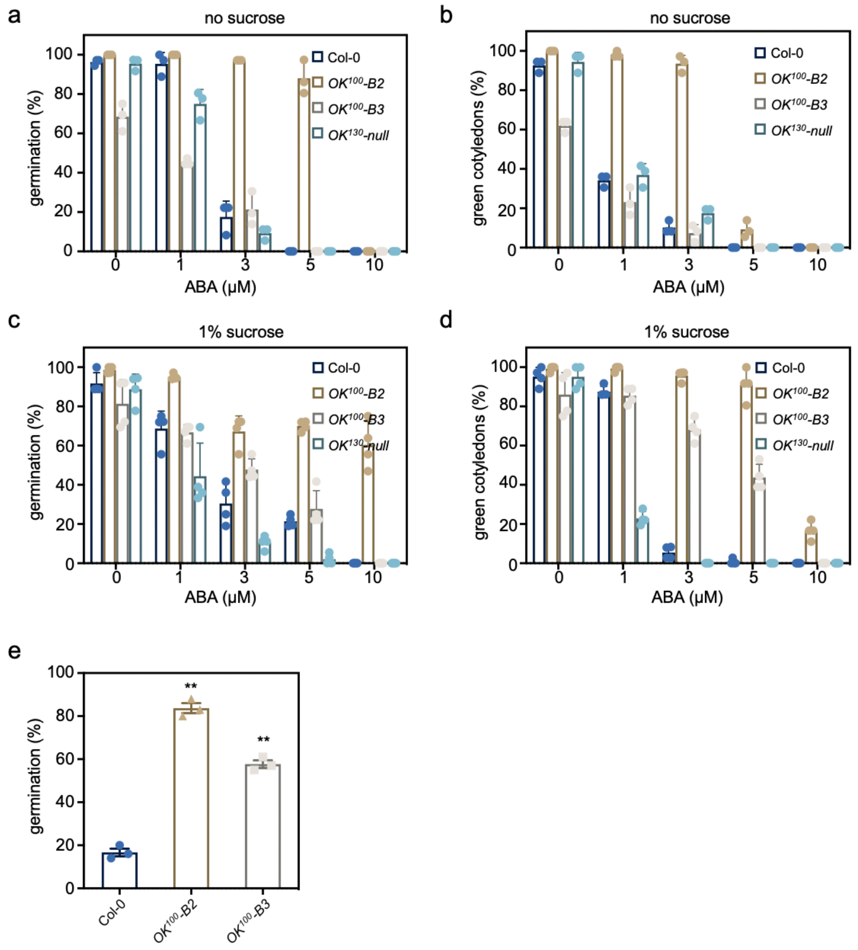

Supplementary Figure 4. Germination and seed dormancy assays of OK^{100}-B3 and OK^{100}-B2 high-order mutants.

a The germination rate of seeds after 3 days of germination and growth on 1/2 MS medium without sucrose, and containing different concentrations of ABA. Error bars, SEM. Each dot represents one experiment. n = 36 seeds in each experiment. **b** The percentage of seedlings showing green cotyledons after 7 days of germination and growth on 1/2 MS medium without sucrose, and containing different concentrations of ABA. Error bars, SEM. Each dot represents one experiment. n = 36 seeds in each experiment. **c** The germination rate of seeds after 3 days of germination and growth on 1/2 MS medium with 1% sucrose, and containing different concentrations of ABA. Error bars, SEM. Each dot represents one experiment. n = 36 seeds in each experiment. **d** The percentage of seedlings showing green cotyledons after 7 days of germination and growth on 1/2 MS medium with 1% sucrose, and containing different concentrations of ABA. Error bars, SEM. Each dot represents one experiment. n = 36 seeds in each experiment. **e** The germination rate of fresh harvested seeds after 2 days of germination and growth on 1/2 MS medium with 1% sucrose. Error bars, SEM. Each dot represents one experiment. n = 36 seeds in each experiment. Two-tailed paired *t*-tests, ** $p < 0.01$. Source data are provided in Source Data.

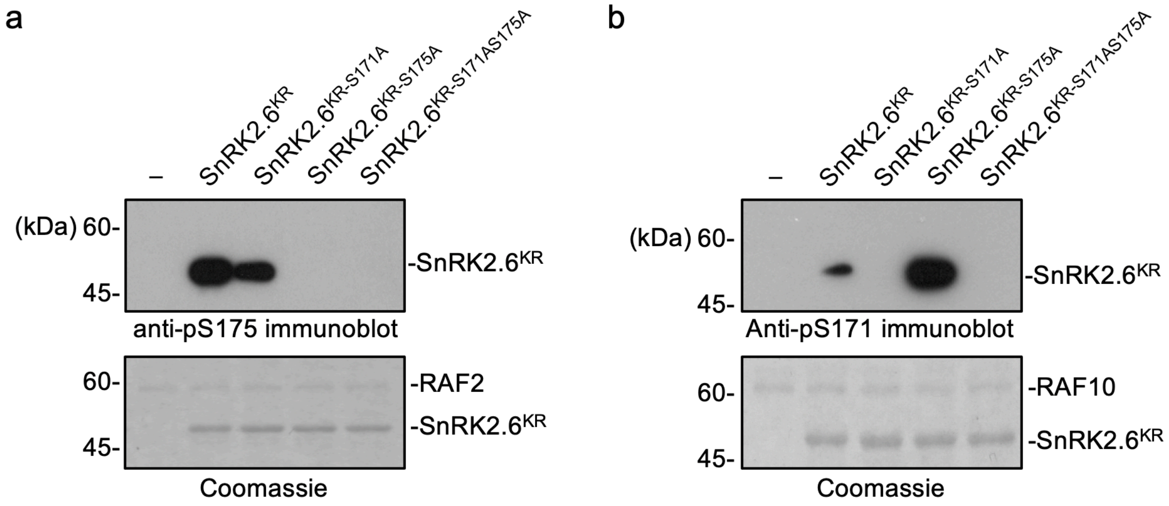

c

```
>AT3G46760.1 LECRK-S.3
  1 MFAGIFLRMG AALRSMYVNS KYEEVREEWE EDYSPQRFSY KALYKATKGF
 51 KESELFGTEA NGTVYKGKLS SNAQIAVKRV SLDAEQDTKH LVSQIVGIGK
101 LRHKNLVQLL GYCRRKGELL LVYDYMPYGN LDDFLFNEER PNLSWSQRFH
151 IIKGVASALL YLHEQIVLHR DVKAANVLLD EDLNGRLDYG LARFGTNRNP
201 MLGSVGYVAP ELIITGMPTT KADVYSFGAL LLEFACGRMF IEYPGKPEEF
251 NLISWVCQCW KRGNLVGARD ARLEGDYVCK EIEMVLKLGL LCAQYNPEDR
301 PSMSQVVNYL EGNDVLPEMP PDTPGISIPT PYHEVLA
```

Supplementary Figure 5. Validation of pS171 and pS175 antibodies.
a Validation of anti-pS175 antibody. The wild type and mutated recombinant HIS-SnRK2.6^{KR} proteins were incubated with recombinant GST-RAF2-KD in the presence of ATP. The resulting products were separated by SDS-PAGE and immunoblotted with anti-pS175 antibody (upper). Coomassie staining (lower) was used to show the loading of purified GST-RAF2-KD and HIS-SnRK2.6^{KR} proteins. **b** Validation of anti-pS171 antibody. The wild type and mutated recombinant SnRK2.6^{KR} proteins were incubated with recombinant GST-RAF10-KD in the presence of ATP. The resulting products were separated by SDS-PAGE and immunoblotted with anti-pS171 antibody (upper). Coomassie staining (lower) was used to show the loading of purified GST-RAF2-KD and HIS-SnRK2.6^{KR} proteins. Images shown are representative of at least two independent experiments. **c** A protein kinase AT3G46760.1 with two phosphosites, T215 and T219 (highlighted), was identified by mass spectrometry analysis of immunoprecipitation using anti-pS171 antibody and untreated wild type seedlings, might be corresponding to the non-specific band in the immunoblot. Source data are provided in Source Data and Supplementary Data2.

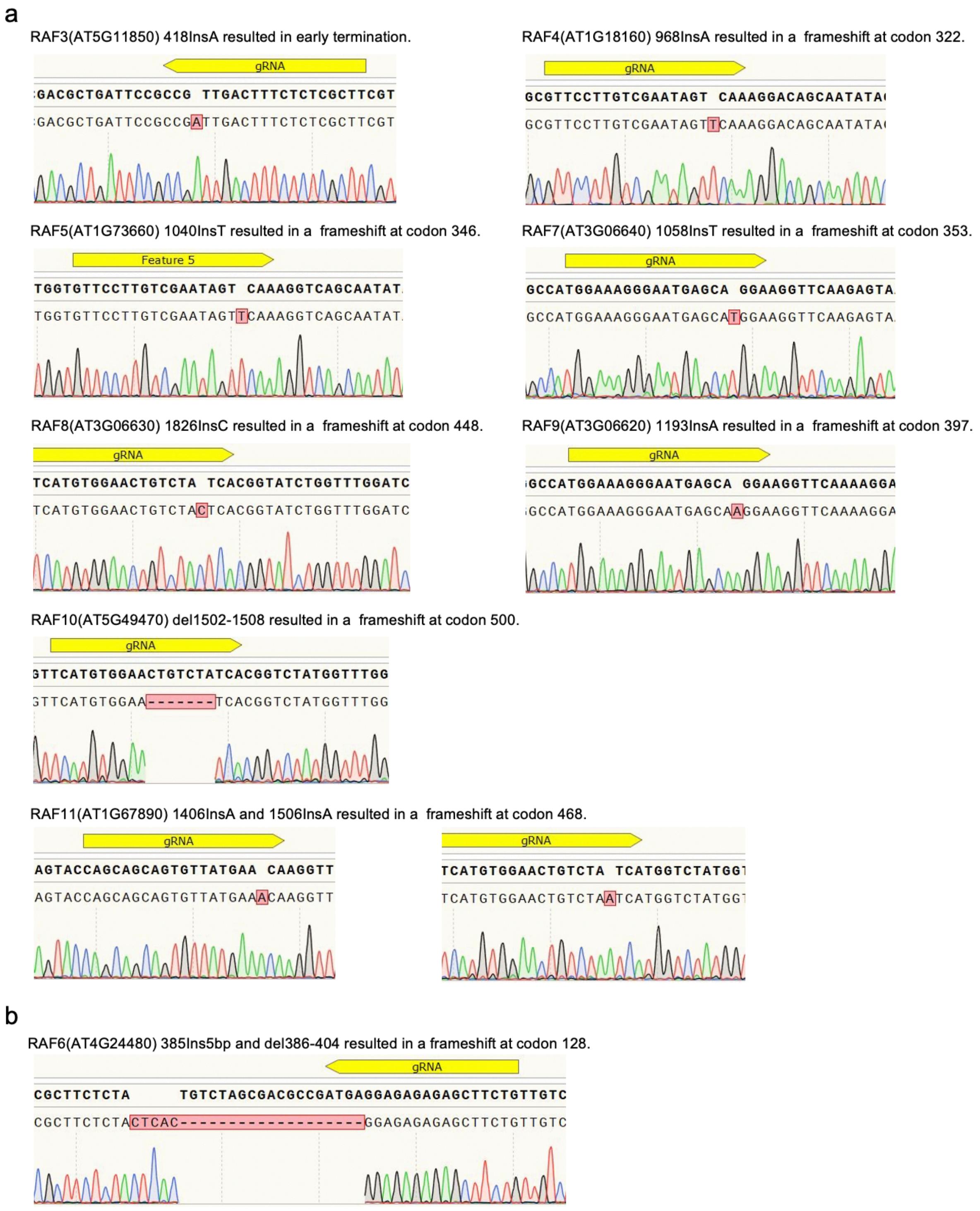

Supplementary Figure 6. Mutations of RAF genes in the OK[100]-oct and OK[100]-nonu high-order mutants.

a Sequencing results showing the mutations in *RAF3*, *RAF4*, *RAF5*, *RAF7*, *RAF8*, *RAF9*, *RAF10*, and *RAF11* genes in the *OK[100]-oct* mutant. **b** Sequencing result showing an additional mutation in *RAF6* in the *OK[100]-nonu* mutant.

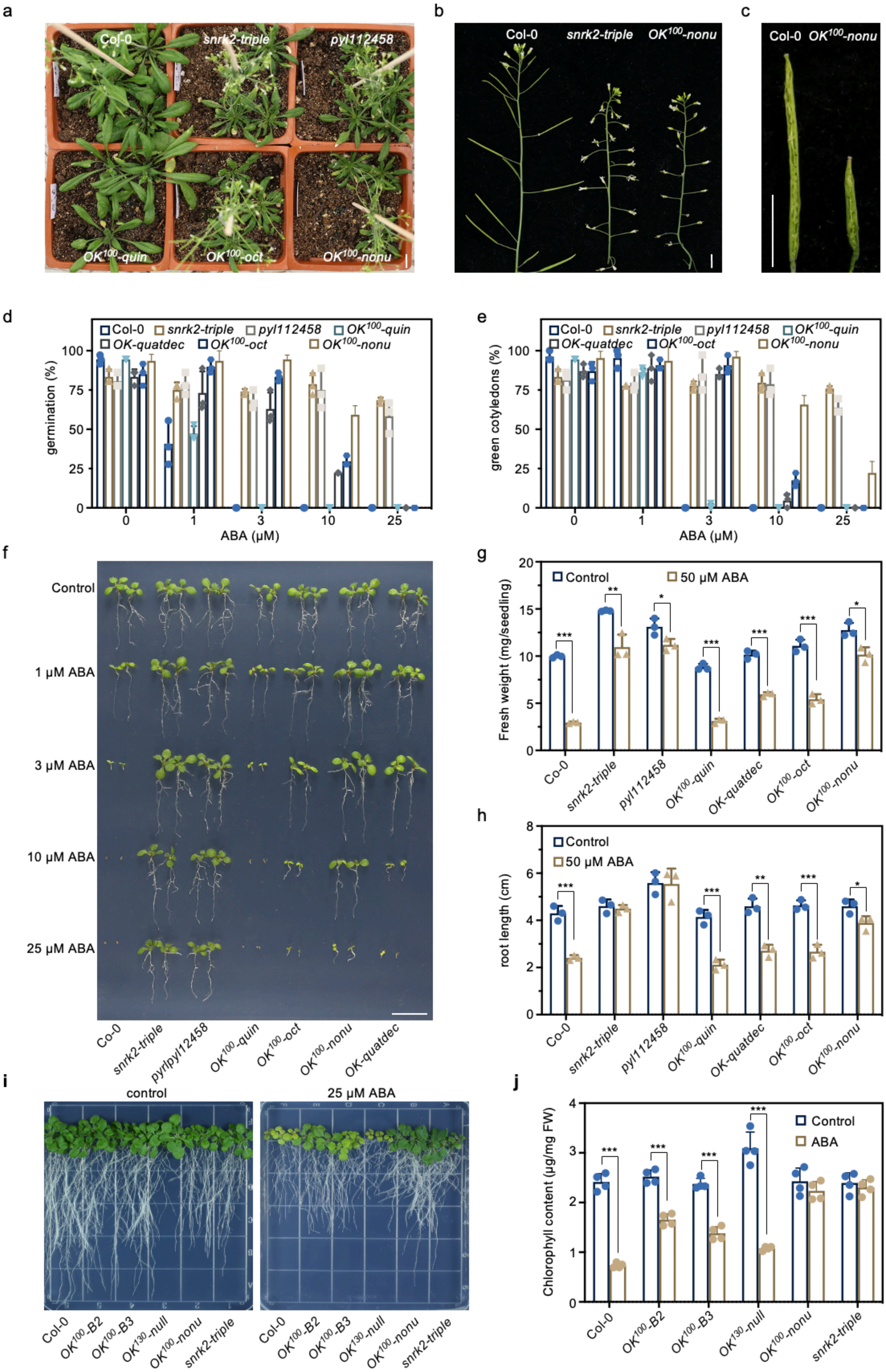
a
Col-0
snrk2-triple
pyl112458
OK100-quin
OK100-oct
OK100-nonu
b
Col-0
snrk2-triple
OK100-nonu
c
Col-0
OK100-nonu
d
Col-0
snrk2-triple
pyl112458
OK100-quin
OK-quatdec
OK100-oct
OK100-nonu
germination (%)
ABA (μM)
e
green cotyledons (%)
ABA (μM)
f
Control
1 μM ABA
3 μM ABA
10 μM ABA
25 μM ABA
Co-0
snrk2-triple
pyrlpyl12458
OK100-quin
OK100-oct
OK100-nonu
OK-quatdec
g
Control
50 μM ABA
Fresh weight (mg/seedling)
h
root length (cm)
i
control
25 μM ABA
Col-0
OK100-B2
OK100-B3
OK130-null
OK100-nonu
snrk2-triple
j
Control
ABA
Chlorophyll content (μg/mg FW)

Supplementary Figure 7. Growth and ABA response phenotypes of the *OK*^{100} high-order mutants.

a Photograph of 6-week-old plants of wild type and mutants in soil; bar = 1 cm. **b** Photograph of inflorescences from the wild type, *snrk2-triple*, and *OK*^{100}*-nonu* mutants; bar = 1 cm. **c** Photographs of silique from the wild type, *snrk2-triple*, and *OK*^{100}*-nonu* mutants; bar = 1 cm. **d** The germination rate of seeds after 3 days of germination and growth on 1/2 MS medium containing different concentrations of ABA. Error bars, SEM. Each dot represents one experiment. n = 36 seeds in each experiment. **e** The percentage of seedlings showing green cotyledons after 7 days of germination and growth on 1/2 MS medium containing different concentrations of ABA. Error bars, SEM. Each dot represents one experiment. n = 36 seeds in each experiment. **f** Photographs of seedlings after 10 days germination and growth on medium containing indicated concentration of ABA; bar = 1 cm. **g** The fresh weight of the seedlings in (**Fig. 4e**). Error bars, SEM. Each dot represents one experiment. n = 10 seedlings in each experiment.). Two-tailed paired *t*-tests, * $p < 0.05$, ** $p < 0.01$, *** $p < 0.001$. **h** Quantitative measurement of the root length of the seedlings in (**Fig. 4e**). Error bars, SEM. Each dot represents one experiment. The number of seedlings in each experiment indicated in Source data. Two-tailed paired *t*-tests, * $p < 0.05$, ** $p < 0.01$, *** $p < 0.001$. **i** Photographs of seedlings growing 14 days after transfer to 1/2 MS medium containing 1% sucrose and 25 µM ABA. **j** The chlorophyll content of the seedlings growing 14 days after transfer to 1/2 MS medium containing 25 µM ABA. Error bars, SEM. Each dot represents one experiment. Two-tailed paired *t*-tests, *** $p < 0.001$. Source data are provided in Source Data.

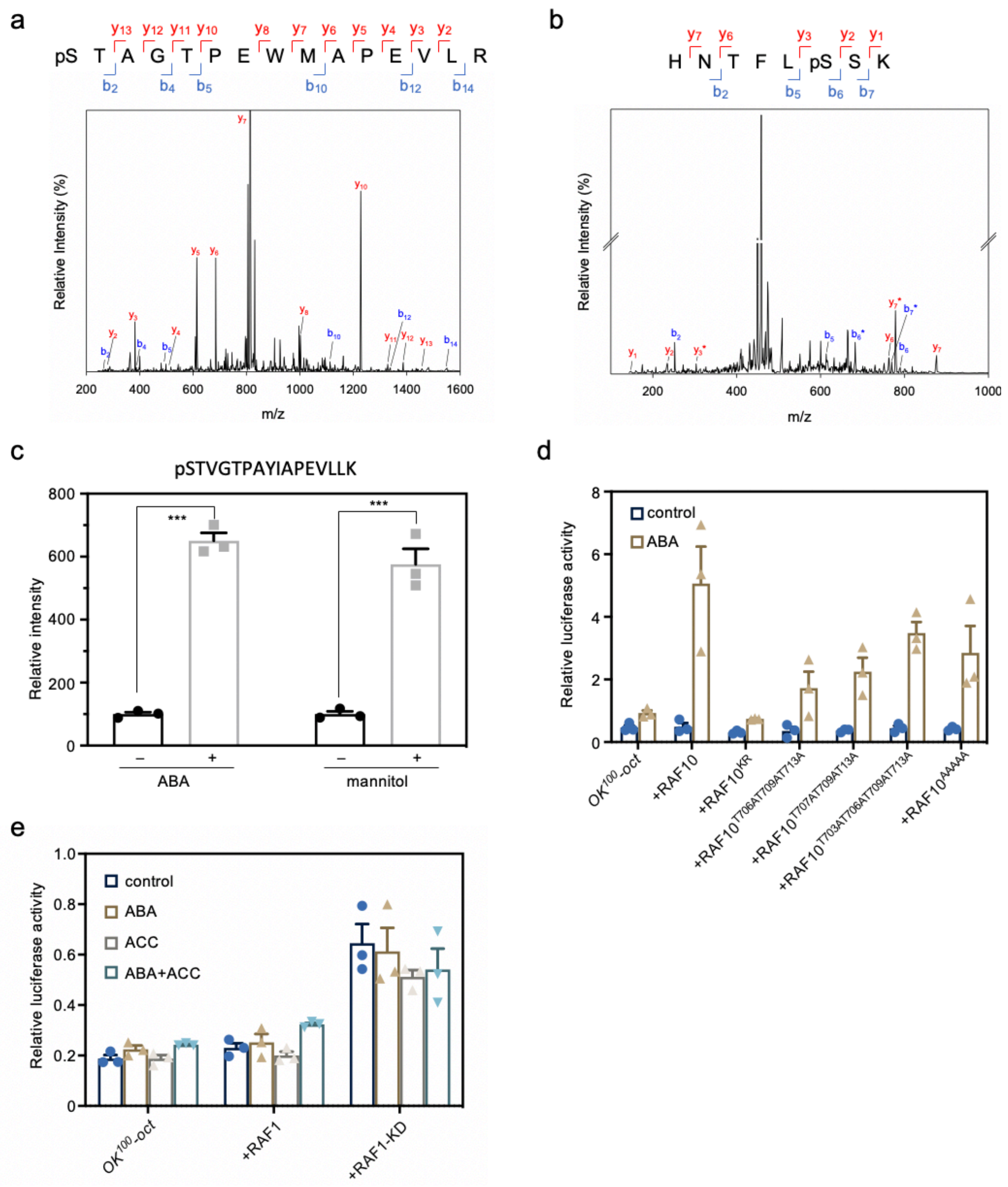

Supplementary Figure 8. Phosphosites in the activation loop of B2 and B3 RAFs.

a The MS/MS spectrum showing the phosphopeptide pSTAGTPEWMAPEVLR in RAF2/EDR1 and RAF3. **b** The MS/MS spectrum showing the phosphopeptide HNTFLpSSK in RAF2/EDR1. **c** The phosphorylation of the Ser175 in SnRK2.6 upon mannitol and ABA treatment. The relative intensity of the phosphopeptide was obtained from previous phosphoproteomics results. Error bars, SEM (n = 3 individual transfections). Two-tailed paired *t*-tests, *** $p < 0.001$. **d** Activation of the *RD29B-LUC* reporter gene by wild type and the non-phosphorylatable mutant of Ser760, Ser763, Ser764, Ser766, and Ser770 in RAF10 in transient reporter gene expression in protoplasts of *OK^{100}-oct*. RAF10^{K515R} (RAF10^{KR}), a kinase-dead form of RAF10, is used as a control. Error bars, SEM (n = 3 individual transfections). **e** Activation of the *RD29B-LUC* reporter gene by co-transfection of full-length RAF1 and RAF1-KD in the protoplasts of *OK^{100}-oct*. Error bars, SEM (n = 4 individual transfections). Source data are provided in Source Data.
