# Supplementary material for: Initiation and amplification of SnRK2 activation in abscisic acid signaling
Source: Nat Commun. 2021 Apr 28;12:2456. doi: 10.1038/s41467-021-22812-x (PMC8080645; doi:10.1038/s41467-021-22812-x)
Supplement: Supplementary file 3 — Descriptions of Additional Supplementary Files [file 41467_2021_22812_MOESM3_ESM.pdf]

Descriptions of Additional Supplementary Files

Supplementary Dataset 1
Description: The trans- and autophosphorylation sites in SnRK2.6M94G.

Supplementary Dataset 2
Description: The mass spectrometry analysis of immunoprecipitation using anti-SnRK2.6-pS171 antibody.

Supplementary Dataset 3
Description: DEGs in the OK100-oct mutant.

Supplementary Dataset 4
Description: Enriched GO of the DEGs exclusively induced in the OK100-oct.

Supplementary Dataset 5
Description: The phosphosites of RAFs in wild type seedlings with or without ABA treatment.
Supplementary Dataset 6. Primers used in this study.
